# Discovery and Validation of Key Biomarkers Based on Immune Infiltrates in Alzheimer’s Disease

**DOI:** 10.3389/fgene.2021.658323

**Published:** 2021-07-01

**Authors:** Zhuohang Liu, Hang Li, Shuyi Pan

**Affiliations:** ^1^The Fifth Clinical Medical College of Anhui Medical University, Beijing, China; ^2^Department of Hyperbaric Oxygen, Sixth Medical Center, Chinese PLA General Hospital, Beijing, China

**Keywords:** genetic correlation, Alzheimer disease, immune infiltration, bioinformatics analysis, GSEA

## Abstract

**Background:**

As the most common neurodegenerative disease, Alzheimer’s disease (AD) leads to progressive loss of cognition and memory. Presently, the underlying pathogenic genes of AD patients remain elusive, and effective disease-modifying therapy is not available. This study explored novel biomarkers that can affect diagnosis and treatment in AD based on immune infiltration.

**Methods:**

The gene expression profiles of 139 AD cases and 134 normal controls were obtained from the NCBI GEO public database. We applied the computational method CIBERSORT to bulk gene expression profiles of AD to quantify 22 subsets of immune cells. Besides, based on the use of the Least Absolute Shrinkage Selection Operator (LASSO), this study also applied SVM-RFE analysis to screen key genes. GO-based semantic similarity and logistic regression model analyses were applied to explore hub genes further.

**Results:**

There was a remarkable significance in the infiltration of immune cells between the subgroups. The proportions for monocytes, M0 macrophages, and dendritic cells in the AD group were significantly higher than those in the normal group, while the proportion of some cells was lower than that of the normal group, such as NK cell resting, T-cell CD4 naive, T-cell CD4 memory activation, and eosinophils. Additionally, seven genes (ABCA2, CREBRF, CD72, CETN2, KCNG1, NDUFA2, and RPL36AL) were identified as hub genes. Then we performed the analysis of immune factor correlation, gene set enrichment analysis (GSEA), and GO based on seven hub genes. The AUC of ROC prediction model in test and validation sets were 0.845 and 0.839, respectively. Eventually, the mRNA expression analysis of ABCA2, NDUFA2, CREBRF, and CD72 revealed significant differences among the seven hub genes and then was confirmed by RT-PCR.

**Conclusion:**

A model based on immune cell infiltration might be used to forecast AD patients’ diagnosis, and it provided a new perspective for AD treatment targets.

## Introduction

As a neurodegenerative disease, Alzheimer’s disease (AD) is usually accompanied by cognitive dysfunction and memory impairment, and even affects speech and motor ability (Ono and Tsuji, 2019). Over 47 million people are estimated to suffer from dementia, and AD as the most common form accounts for 60–80% of them (Barker et al., 2002; Anand et al., 2017). The pathologic changes of AD remain the gold standard for diagnosis characterized by brain atrophy, ventricular enlargement, extracellular amyloid plaques, intracellular neurofibrillary tangles, and so on (Piguet et al., 2009; Serrano-Pozo et al., 2011). However, the preclinical stage of AD patients barely meets the diagnostic criteria until pathology can be found in the postmortem brain. Although amyloid and tau biomarkers can be detected before structural brain changes, there is still a lack of effective diagnosis in the pre-dementia phase.

The risk factors of development for AD diagnosis are important, of which age is dominant (Currais et al., 2019). As the population ages, the prevalence of AD is increasing (Lee et al., 2019). Studies have shown that for people over 60, the incidence doubles per 6.3 years. Besides, family history, Down syndrome, and cerebrovascular disease are also important risk factors (DeTure and Dickson, 2019). These different multifaceted contributors can increase risk for the disease; nevertheless, the genetic factor warrants special emphasis with a higher heritability percentage of about 60–80% (Gratuze et al., 2018).

Familial AD is closely related to mutations in amyloid precursor protein (APP), presenilin 1 (PSEN1), or PSEN2 genes (Ryman et al., 2014). Additionally, the apolipoprotein E4 gene (APOE4) is one of the greatest risks in progressing AD (Ballard et al., 2016; Lane et al., 2018). The genome-wide association studies indicated that TREM2, ADAM10, and PLD3 all affect APP and tau, and regulate directly known functions, including immune response, cholesterol metabolism, and endocytosis (Chouraki and Seshadri, 2014; Karch and Goate, 2015; Jansen et al., 2019). Growing evidence suggests that Alzheimer’s disease pathogenesis is strongly associated with immunological mechanism (Shi and Holtzman, 2018; Webers et al., 2020). The binding of AD-related proteins and receptors on microglia can initiate an innate immune response and subsequently cause disease deterioration (Sarlus and Heneka, 2017). Previous studies showed that amyloid plaque burden was negatively correlated with the levels of the immune biomarker, anti-amyloid-β (anti-Aβ) autoantibodies in AD, which links the disease progression and Aβ accumulation (Kellner et al., 2009; Storace et al., 2010). The dysfunction of central and peripheral immune systems was associated with several differentially expressed immune markers including CD3, CD4, CD7, CD28, and CD56 in AD. It has been shown that TREM2 gene mutations greatly increase the risk of AD, and phagocytosis of microglia is mediated with receptors on microglia and astrocytes (Heneka et al., 2015; Yeh et al., 2017). The phagocytic capability for Aβ is weakened, which may be implicated in MGAT-III and Toll-like receptor-3 (Avagyan et al., 2009). Increasing evidence suggests that the rare variants of TREM2 can promote the development of amyloid and tau pathologies due to the dysfunction of microglia (Gratuze et al., 2018). Also, TREM2 can participate in the interaction with other biomarkers including APOE (Qin et al., 2021). Collectively, Trem2 acts as the immune checkpoint and the scavenger receptor to clear harmful stimuli in the regulation of neurocyte survival (Hickman et al., 2018). Risk genes consolidate a role of the immune system in AD and have been identified relating to complements and various immune cells, including microglia, monocytes, and B and T lymphocytes (Jevtic et al., 2017). Up to now, there is no disease-modifying therapy for Alzheimer’s disease. Therefore, finding novel risk factors and targeting these immune mechanisms are needed to develop future therapeutic approaches for Alzheimer’s disease.

To explore and identify potential biomarkers of AD, gene chips were obtained from the Gene Expression Omnibus database. We then identified hub genes through LASSO and BORUTA analysis. The combined use of two algorithms for screening biomarkers will make the process more rigorous and standard; finally, identified key biomarkers are strongly associated with AD. Then the CIBERSORT algorithm was applied to calculate the immune infiltration of AD samples. Moreover, the relationship between 22 immune cells and hub genes was analyzed. The logistic regression model was investigated by ROC and further validated by an external dataset. This is of great significance and the first utilization of a combination of LASSO and SVM-RFE method to reveal the key biomarkers in AD.

## Materials and Methods

### Gene Expression Data Acquisition

The GSE63061 data file was downloaded from the NCBI Gene Expression Omnibus public database (GEO^1^) annotated by GPL10558 as a Series Matrix File. The file contains data related to 273 groups of patients’ expression profiles, including 134 normal groups and 139 AD patients. Meantime, the GSE85426 Series Matrix File was obtained from the GEO public database. The annotation file is GPL14550, which consists of 180 groups of patients’ expression profile data involving 90 cases of normal group and 90 cases of AD patients.

### Analysis of Immune Cell Infiltration

The CIBERSORT is widely used in evaluating the type of immune cell in the microenvironment. The tool is based on the principle of linear support vector regression to perform deconvolution analysis on the expression matrix of immune cell subtypes. It contains 547 biomarkers, and 22 phenotypes of human immune cell, covering plasma cells, B cells, T cells, and myeloid cell subsets, have also been defined. Using the CIBERSORT algorithm, this study analyzed the AD patients’ data and quantified the relative proportion of 22 infiltrating immune cells. Furthermore, this study performed Spearman correlation analysis on immune cells and gene expression.

### Construction of LASSO Model and SVM-RFE Feature Selection Process

The LASSO logistic regression and the SVM algorithm were used to classify the diagnostic markers of AD. The LASSO analysis was undertaken using the “glmnet” package, the response type was set as binomial, and the alpha was set as 1. Besides, as a surveillant machine learning method to support vectors, the Support Vector Machine (SVM) finds the best variables by deleting the feature vectors generated by the SVM. The SVM classifier from R package e1071 was adopted for classification analysis of the selected biomarkers in the diagnosis of AD; *k* = 5 was the setting for the *k*-fold cross validation, and the parameter of halving above was identified as 100.

### Gene Set Enrichment Analysis (GSEA)

Patients are differentiated into two groups (the high expression group and the low expression group) based on the expression level of seven pivot genes. The GSEA analysis of the two groups was achieved by applying signal pathway differences. Molecular Signature Database v7.0 provided the background gene set data required for this study (MSigDB)^2^. Annotated gene sets were used to distinguish subtypes by the identified differentially expressed genes. We computed the consistency *P*-value for each gene set, and *P*-values less than 0.05 were considered significantly enriched. Subsequently, significantly enriched gene sets were ranked. The association between disease type and biological processes was analyzed by using GSEA.

### Semantic Similarity GO Annotations

In this paper, we incorporated GO-based semantic similarity and ranked the proteins by the functional similarity of protein–protein interactions. The relationship between semantic similarity in the GO annotation and the correlation of the gene expression profile was verified (Sevilla et al., 2005). It provides a basis for the functional comparison of gene products, so it has been widely used in bioinformatics, such as protein–protein interaction analysis (Jain and Bader, 2010), pathway analysis (Guo et al., 2005), and gene function prediction (Tedder et al., 2010). Here, we measured the functional similarity between proteins. The functional similarity is defined as the geometric mean of the semantic similarity of GO in terms of the molecular function (MF) and the biological process (BP). To quantify the correlations between proteins, the biological function and the pathway were included. The semantic similarity of interacting proteins in MF and BP is performed by using the GO SemSim software package of Wang’s method (Yu et al., 2010). It was carried out in a more accurate method that involved GO topology (Wang et al., 2007). The geometric average of semantic similarity in MF and BP algorithms was used to further estimate the functional similarity.

### Co-expression Analysis of the Hub Genes

Correlation analysis was performed using the corrplot and circlize package in R software. Pearson correlation on the hub gene expression was carried out using the package corrplot (version 0.84) in R. The circos plot was generated through the circlize package. The correlation coeÿcient was depicted by the colors “red” and “green.” Red denotes a positive correlation, whereas green indicates a negative correlation. The darker the color and the thicker the string, the stronger the correlation.

### Differential Expression Analysis

To explore the relationship between hub genes and the development of AD, we performed the differential expression analysis for hub genes in different types of brain tissues using the AlzData. AlzData^3^ is a comprehensive AD high-throughput omics database (Xu et al., 2018). AlzData can be used as an in-depth integration system, integrating different levels of data for disease characterization. It is comprised of genomics (GWAS and whole exome sequencing), transcriptome, proteomics, and functional genomics data. In addition, the study also further calculated the CFG (convergent functional genomic) score (Supplementary Table 1) of key genes.

### RT-PCR Validation of the Hub Genes

The serum samples of six patients without AD and six patients with AD were acquired for RT-PCR verification in order to verify the predictive analysis results. The Ethics Committees or institutional review board of the Sixth Medical Center of Chinese PLA General Hospital approved this protocol. Based on the manufacturer’s instructions, this study used a TRIzol reagent to extract total RNA (Invitrogen, 10296028). RNA quality (RNA integrity number, RIN) was determined by Agilent 2100 Bioanalyzer analysis (Agilent Technologies, CA, United States), and RIN ≥ 8 for subsequent experiments. RNA samples from total RNA were reverse-transcribed to cDNA, and RT-PCR was performed using the SuperScript III RT (ABI-Invitrogen, 11752050). β-Actin was used as an internal normalization standard. Relative mRNA expression was calculated using the ΔΔCT method. Primers were as described in the Table 1.

**TABLE 1:** Primer information.

**Target name**	**Primer**	
β-Actin	F	GACAGGATGCAGAAGGAGATTACT
	R	TGATCCACATCTGCTGGAAGGT
ABCA2	F	CATCGCCATCTTCATCATCC
	R	TTGAGCATGTCCCACTCGAA
CREBRF	F	GGAACAAACTCTGATGC
	R	CATTTAGTTGGCTGTTCAC
CD72	F	CATCTCCAGCAGGTTAGGACA
	R	CGGGCACTTGAACATTCTC
CETN2	F	AAAGAATGAGACCTAAGCC
	R	CTTTAACATCTATAGTGCGAG
KCNG1	F	CCGAGTTCACCTGCATCCC
	R	CCATAGCCCACCGTCCTC
NDUFA2	F	TCTCATCCACTTATGTC
	R	CTTCACATCGGAGCCT
RPL36AL	F	CCTACCTAAAACCCGAAT
	R	TTCTTTGTGGTCTTAGCGTT

### Statistical Analysis

*T*-test was used for measurement data (expressed as a mean ± SEM), while chi-squared test was used for categorical data (presented as percentages). The logistic regression algorithm was used to build the predictive model. All statistical analyses were performed by R (version 3.6), and GraphPad Prism 8. All experiments were done in triplicate. Significance was defined as *P* < 0.05 for two-sided tests.

## Results

### Identification of Hub Genes

**FIGURE 1:**
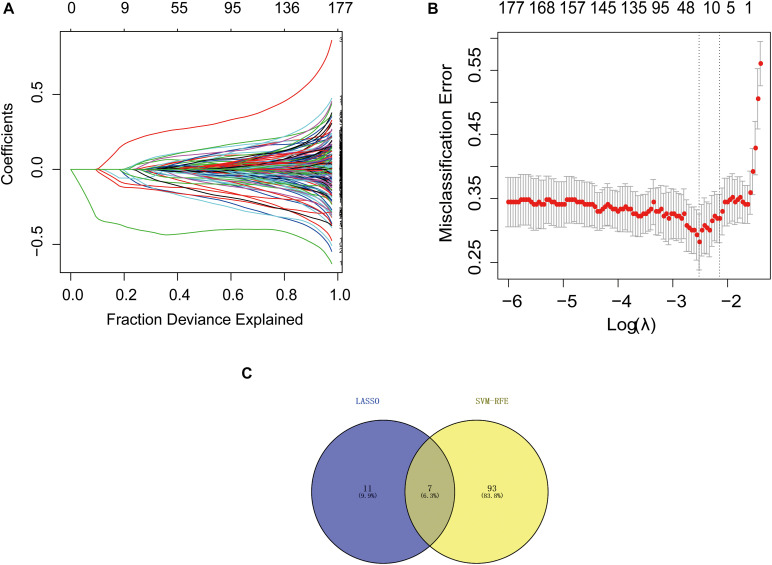
Selection of diagnostic biomarkers and identification of hub genes. **(A)** LASSO coefficient profiles of the 11 genes that met the prognostic criteria initially. **(B)** The misclassification error in the jackknife rates analysis. **(C)** Venn diagram of genes extracted from LASSO and SVM-RFE methods. LASSO, least absolute shrinkage and selection operator; SVM, support vector machine; RFE, recursive feature elimination.

We downloaded the GSE63061 dataset from the NCBI GEO public database. The total number of patients was 273 (AD group, 139; control group, 134). To explore the biomarkers of AD, we performed feature screening through LASSO regression. The results from the LASSO regression showed that 18 genes were identified as signature genes in AD. On the other hand, we use the SVM-RFE algorithm to evaluate the characteristic genes in AD. The data illustrated that a total of 7 differentially expressed genes were acquired from the intersection of the top 100 scoring genes and the characteristic genes selected by the LASSO regression algorithm. The intersection genes were used as the core genes for subsequent research (Figure 1).

### Immune Infiltration Analyses

**FIGURE 2:**
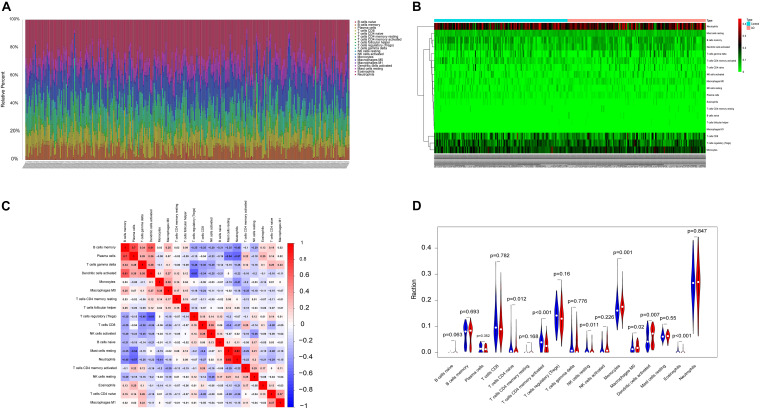
The landscape of immune infiltration between AD and normal controls. **(A,B)** The box-plot diagram indicating the relative percentage of different types of immune cells and the heat map summarizing the score of immune infiltration between AD patients and non-AD patients. **(C)** The heat map shows the correlation in infiltration of innate immune cells by CIBERSORT. **(D)** The difference of immune infiltration between AD (red) and normal (blue) controls. (*P*-values < 0.05 indicated statistical significance).

The microenvironment is composed of immune cells, inflammatory factors, extracellular matrix, and various growths, and it has an important impact on clinical treatment sensitivity and disease diagnosis. Through studying the relationship between immune infiltration and the hub gene in the AD dataset, the potential molecular mechanisms of core genes influencing the AD progression have been further explored. The results indicated that the fractions for monocytes, M0 macrophages, and dendritic cells in the AD group were remarkably higher compared with those of the normal patients, while the fractions of many cells are lower than those of the normal patients, such as T-cell CD4 memory activation, T-cell CD4 naive, eosinophils, and NK cell resting. The interaction between the immune cells is shown in Figure 2.

### Analysis of Hub Genes and Immune Infiltration

**FIGURE 3:**
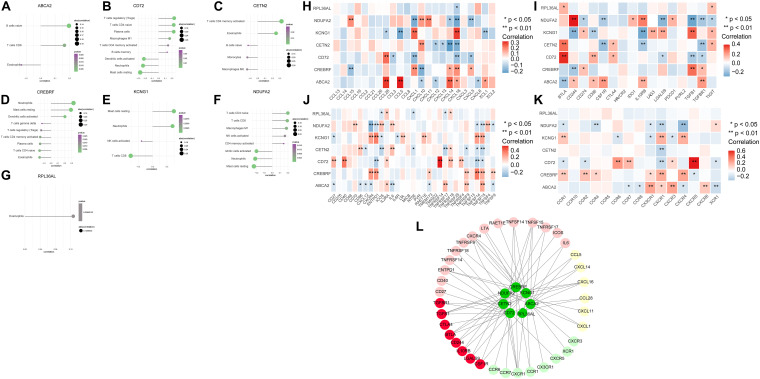
The association between the hub genes and immune cell infiltration. **(A–G)** Correlations between hub genes and the infiltration level. **(H–K)** Correlation between hub genes and chemokines, immunosuppressive factors, immunostimulatory factors, and immune receptors. **(L)** Protein–protein interaction plot of hub genes and immune-related molecules.

To explore further the relationship between core genes and immune cells, the results indicated that ABCA2 was positively correlated with B cells naive. Besides, CREBRF was positively correlated with neutrophils, dendritic cells activated, and mast cells resting. The CD72 was positively correlated with T regulatory cells (Tregs), T-cell CD4 naïve, and plasma cells but negatively correlated with dendritic cells activated, neutrophils, and mast cells resting. CETN2 had a positive relationship with T cells, eosinophils, and CD4 memory activation. KCNG1 was positively correlated with mast cells resting and neutrophils, while it was negatively correlated with T-cell CD8. There was a positive correlation between NDUFA2 and T-cell CD4 naive, and it was negatively associated with dendritic cells activated, neutrophils, and mast cells resting (Figures 3A–G). We next obtained the correlations between these seven key genes and different immune factors including immunosuppressive factors, immunostimulatory factors, chemokines, and receptors from the TISIDB database. The graphs of the correlational relationships between immune factors and AD core genes were constructed (Figures 3H–K). We selected immune factors related to the hub gene (the average correlation coeÿcient greater than 0.2) and used STRING and Cytoscape to construct an interaction network (Figure 3L). These analyses confirmed that key genes were closely related to the level of immune cell infiltration and played a crucial role in the immune microenvironment.

### Analyses of Biological Processes and Core Pathways Enriched for Genes in AD

**FIGURE 4:**
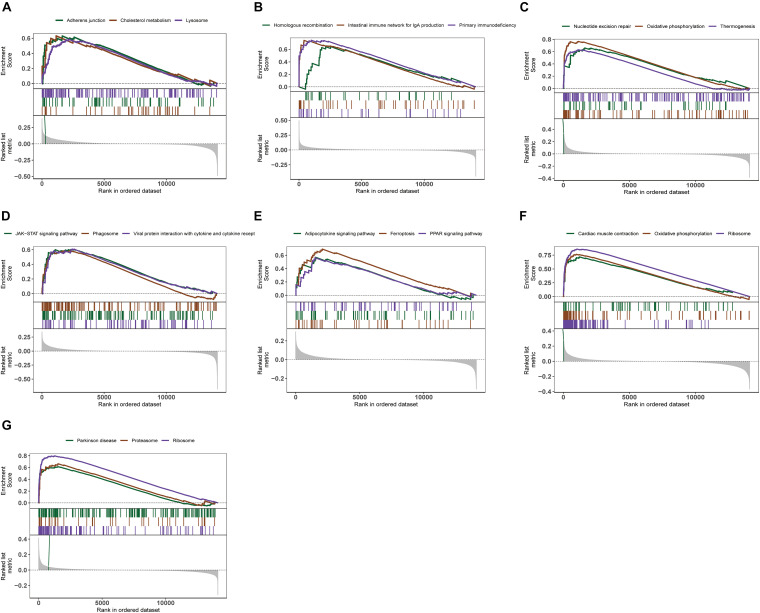
Enrichment analysis of pathway and gene ontology (GO) involved hub genes. **(A–G)** Gene Set Enrichment Analysis of ABCA2, CREBRF, CD72, CETN2, KCNG1, NDUFA2, and RPL36AL.

**FIGURE 5:**
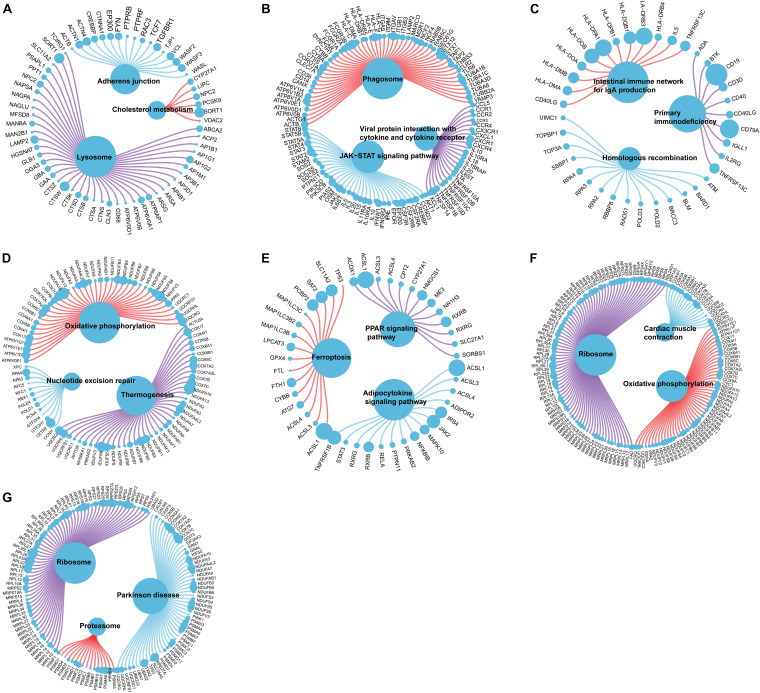
Molecular regulatory mechanism of core gene-related pathway and GO functional enrichment analyses. **(A–G)** GSEA-related ccgraph plot of ABCA2, CREBRF, CD72, CETN2, KCNG1, NDUFA2, and RPL36AL.

**FIGURE 6:**
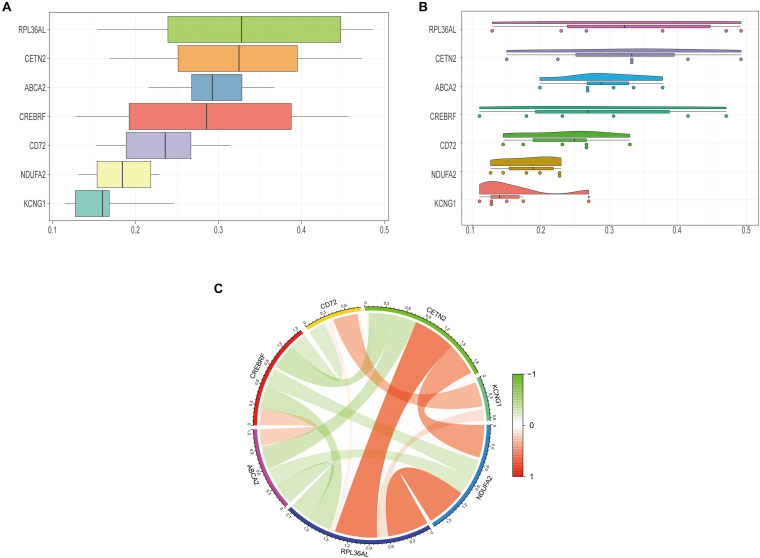
Closeness score of semantic similarities between GO terms and coexpression analysis of hub genes. **(A)** GO semantic similarity box plot of core genes. **(B)** Raincloud plot of relatedness of the GO terms. **(C)** The circos diagram depicts Pearson correlations between hub genes.

We further explored the specific signaling pathways involved in the seven core genes, and explore the potential molecular mechanisms of them that affect the progression of AD. GSEA results showed that the pathway involved in the high expression of ABCA2 is cholesterol metabolism. Meanwhile, the pathway involved in the high expression of KCNG1 is the adipocytokine signaling pathway. The pathways involved in the high expression of CETN2 are oxidative phosphorylation and thermogenesis, all related to energy metabolism. It suggested that these three core genes may participate in the progression of AD by influencing the body’s metabolism. Concurrently, high expressions of NDUFA2 and RPL36AL are related to the ribosome pathway. It suggested that both of them can affect the progression of AD by regulating the function of the ribosome (Figure 4). The molecular interaction network between each pathway is shown in Figure 5. Also, to further identify the core genes that play a key role in AD, we ranked the core genes according to the average functional similarity relationship between the proteins. The results demonstrated that RPL36AL, CETN2, and ABCA2 are the top 3 molecules based on the GO similarity score in AD; their median scores were around 0.35, 0.35, and 0.30, respectively. Besides, the rest of them were under 0.3 remarkably (Figures 6A,B). Correlation analysis of seven hub genes was indicated using the circle diagram in Figure 6C. Furthermore, RPL36AL, CETN2, KCNG1, and NDUFA2 displayed closely positive relationships, but the remaining three genes mainly revealed negative associations with each other. The mechanism remains to be further explored.

### Logistic Regression Is Potential to Build a Core Gene Correlation Model

**FIGURE 7:**
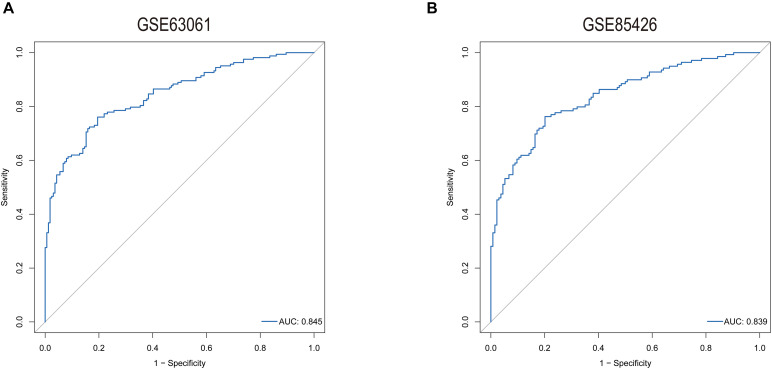
ROC curves for evaluating the accuracy of logistic regression analysis of training set [**(A)**, GSE63061] and validation [**(B)**, GSE85426].

The predictive model was constructed by using the logistic regression algorithm. The results showed that the predictive model constructed by seven genes had better diagnostic performance, and the area value under the receiver operating characteristic curve (AUC) was 0.845. We further downloaded the dataset GSE85426, of which 90 people had AD and 90 were normal patients. Fivefold cross-validation was performed to further verify the diagnosis model, as external validation data. The results showed that the model had strong stability, and the AUC value was 0.839 (Figure 7).

### The Experiment of Seven Hub Genes

**FIGURE 8:**
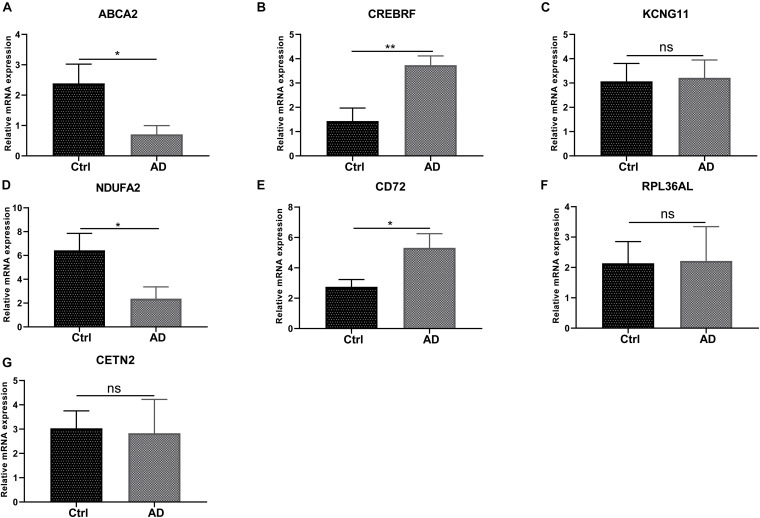
RT-PCR validation of the hub gene between AD and normal controls. **(A-G)** All experiments were carried out three times, and the data were expressed as mean ± SEM (*p < 0.05, **p < 0.01, ns, no significance).

We next conducted RT-PCR experiments to detect the hub genes’ relative expression level in AD and normal control groups. The research data demonstrated that the mRNA expression level of ABCA2 and NDUFA2 in AD decreases compared with that of the controls (*p* < 0.05). Conversely, the opposite results were observed for CREBRF and CD72 (*p* < 0.05, CREBRF; *p* < 0.01, CD72). Besides, there was no significant difference in the levels of CETN2, KCNG1, and RPL36AL between the AD group and the control group (Figure 8). These identified seven genes might function as the potential diagnostic and prognostic biomarkers.

## Discussion

The most common cause of degenerative dementia is Alzheimer’s disease (AD). So far, amyloid plaques and neurofibrillary tangles are its main pathological diagnostic features (DeTure and Dickson, 2019). Nevertheless, detection of AD brain lesions using histopathology is suboptimal, and treatments were currently in a limited pharmacotherapy phase. A growing number of researchers realize that immune infiltration is related to the diagnosis in cancer and other diseases (Barnes and Amir, 2018; Varn et al., 2018; Cai et al., 2020). In this context, the immune system plays an essential role in the etiology of AD. Additionally, immune infiltrates were linked to the target and clearance of amyloid beta-peptide plaques in the brain (Monsonego et al., 2006). The central immune system compositions such as microglia and complements, as well as peripheral immune-related components including monocytes and lymphocytes, can influence the pathology of AD (Jevtic et al., 2017). The previous research demonstrated that C1q is highly correlated with Aβ deposition and activates microglia phagocytosis, which, in turn, leads to synapse loss (Hong et al., 2016). In addition to C1q, elevated Aβ burden can be mediated through C3, also associated with NF-κB in AD (Ascolani et al., 2012; Shi et al., 2017). Besides, CD33 as pivotal genes of microglia reduces phagocytosis, but TREM2 has a reverse effect (Jiang et al., 2008; Griciuc et al., 2013). Equally, the peripheral immune cells such as monocytes, neutrophils, and lymphocytes can promote neuroinflammation in the progress of AD (Erny et al., 2015). When the monocytes were deleted in a mice model, it will worsen the AD pathology (El Khoury et al., 2007). Except for monocyte, neutrophil was proven that its neutrophil extravasation ability and amyloid pathology were ameliorated by the inhibition of LFA-1 (Zenaro et al., 2015). In absence of certain lymphocytes (functional B and T cells), Rag2 knockout can decrease Aβ levels in PSAPP AD mice brain (Späni et al., 2015). Yet, few studies have systematically screened the biomarkers related to AD immune infiltration and their value in assessing AD immune infiltration.

**FIGURE 9:**
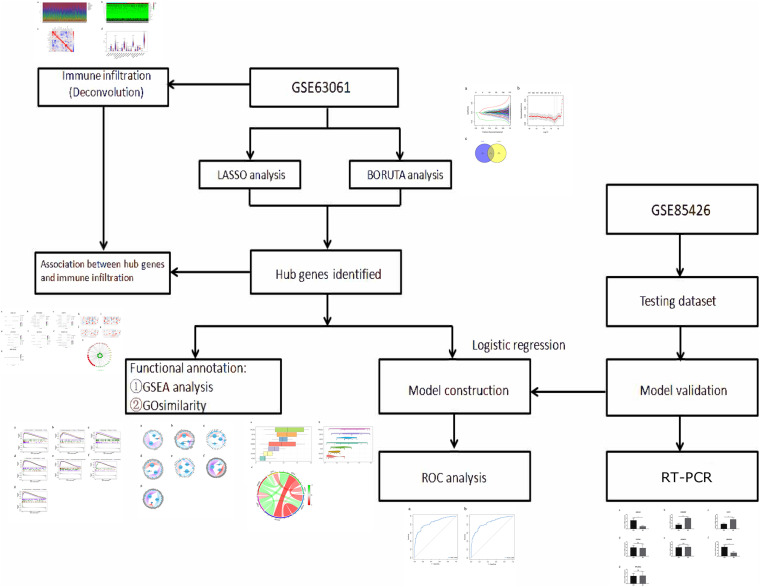
The workflow of analysis.

In the present study, we used LASSO regression analysis and the SVM-RFE method to screen out seven potential genes (ABCA2, CREBRF, CD72, CETN2, KCNG1, NDUFA2, and RPL36AL). Subsequently, the CIBERSORT algorithm performed deconvolution analysis on the immune microenvironment to assess the proportion of the immune cells in AD. What is more, previous research confirmed the effectiveness of the CIBERSORT technique (Dai et al., 2018; Mao et al., 2018; Chang et al., 2020). Besides, the immune cell infiltration scoring model was built by LASSO regression based on 22 immune cells. GO semantic similarity and GSEA analysis annotated the profiles of the seven hub genes. GSEA analysis suggested that there were considerably abundant genes in diverse immune-related biological processes and pathways. The RNA characteristics as indicators of immune cell infiltration in AD patients were identified through adopting this calculation framework to AD patient datasets and immune cell lines. Our research combined with the results of bioinformatics analysis and RT-PCR and showed that there were significant differences in the samples of AD patients containing four genes. We found that ABCA2 and NDUFA2 in the AD group were significantly lower than those in the control group; CREBRF and CD72 mRNA in the AD patients had significantly higher mRNA expression level compared to the control group (Figure 9). Likewise, differential expression analysis of hub genes in various brain cell types was revealed (Supplementary Figure 1 and Supplementary Table 1). ABCA2 is rich in the frontal and temporal areas of the AD brain, involving resistance to reactive oxygen species in the ABCA2 transfected cell line (Chen et al., 2004). In agreement, it is ranked in the top 3 for both GO similarity analyses. Previous reports have pointed out that ABCA2 is a key regulator of endogenous APP expression and AD truncation. In research, CREBRF was proposed as a novel biomarker using weighted gene co-expression network analysis (WGCNA) based on a total of 329 samples (Soleimani Zakeri et al., 2020). Moreover, it has been documented to block autophagy through the CREB3/ATG5 pathway in brain tumor (Xue et al., 2016). Studies showed that CETN2 modulates male mice infertility or dysosmia and affects embryonic development, which is regulated by the FGF/FGFR gene expression (Shi et al., 2015; Ying et al., 2019). CD72 plays a negative role in the regulation of B-cell activation (Davis, 2010). It is reported that the combined effects between Sema4D and CD72 induce immune activation in the central nervous system (Tsubata, 2019). However, the study for CD72 in AD remains poorly understood. Therefore, we call for further research to better elucidate this aspect.

Due to the limited number of samples, there is still a need to confirm these preclinic observations in future clinic studies for novel biomarkers. Moreover, most AD cases have other neurodegenerative comorbidities. Similarly, this phenomenon was also observed in the profile of 1,153 AD pathologically diagnosed patients acquired from Mayo Clinic Brain Bank data (DeTure and Dickson, 2019). Compared to other neurodegenerative diseases, many pathological changes are highly supportive but not specific to AD. Besides, there are similar pathological features between various diseases. For example, Down’s syndrome patients with chromosome 21 trisomy where APP is located can have Alzheimer-like pathology (Kang et al., 1987). APOE4, as a common risk factor of AD, also carries certain morbidity risk for vascular dementia, Down’s syndrome, and brain injury (Verghese et al., 2011). These will need further study.

## Conclusion

In this study, the overlap of the LASSO model and the SVM-RFE algorithm was obtained, and eventually, seven hub genes were recognized. RT-PCR detected these hub genes’ expression levels. To understand AD development, the GSEA and GO analysis of the selected genes supplied a more specific molecular mechanism. Through carrying out the deconvolution algorithms on the patient data in the GEO database, this study revealed that there was distinctness in immune infiltration between the two groups. So far, the relationship between key genes and immune infiltration has been little reported. The mechanism of key genes and immune infiltration-related factors in the diagnosis of AD remains to be explored.

## Data Availability Statement

The raw data supporting the conclusions of this article will be made available by the authors, without undue reservation.

## Ethics Statement

The studies involving human participants were reviewed and approved by the Sixth Medical Center of Chinese PLA General Hospital approved this protocol. The patients/participants provided their written informed consent to participate in this study. Written informed consent was obtained from the individual(s) for the publication of any potentially identifiable images or data included in this article.

## Author Contributions

ZL: conceptualization, methodology, data curation, writing—original draft preparation, visualization, investigation, software, and performed the experiments. HL and SP: conceptualization, methodology, supervision, writing—reviewing and editing. All authors contributed to the article and approved the submitted version.

## Conflict of Interest

The authors declare that the research was conducted in the absence of any commercial or financial relationships that could be construed as a potential conflict of interest.

## Footnotes

1 http://www.ncbi.nlm.nih.gov/geo/

2 http://www.brADd.mit.edu/GSEA/msigdb/

3 http://www.alzdata.org/

## Supplementary Material

Supplementary Figure 1 Differential expression analysis of hub genes in the AlzData database. **(A–F)** Differential expression analysis of ABCA2, CD72, CETN2, KCNG1, NDUFA2, and RPL36AL for various cell types in the brain tissue. No data available about CREBRF in AlzData.

Supplementary Table 1 Convergent functional genomic (CFG) ranking for target genes. 1. eQTL: expression of target gene is regulated by AD genetic variants (genetic variants: IGAP GWAS P-val < 1E-3; eQTL: P-val < 1E-3); 2. GWAS: IGAP P-val < 1E-3; 3. Early_DEG: target gene is differentially expressed in AD mouse models before AD pathology emergence; 4. CFG: total CFG score of target gene, 1 CFG point is added if any of above evidence is significant, CFG point ranges from 0 to 5.
