# Supplementary figures and images for: Discovery and Validation of Key Biomarkers Based on Immune Infiltrates in Alzheimer’s Disease
Source: Front Genet. 2021 Jul 1;12:658323. doi: 10.3389/fgene.2021.658323 (PMC8281057; doi:10.3389/fgene.2021.658323)

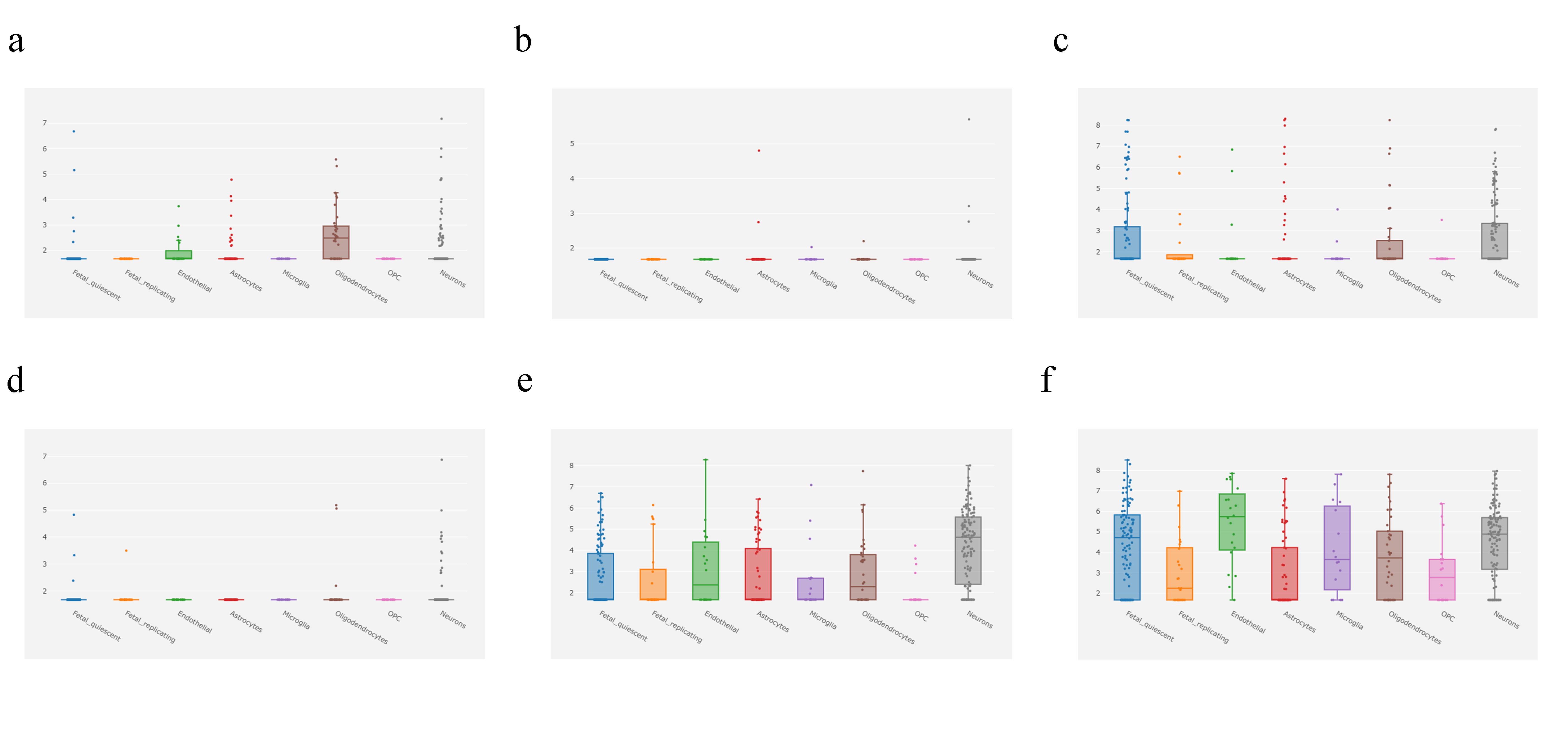

Supplement: Supplementary Figure 1 — Differential expression analysis of hub genes in the AlzData database. (A–F) Differential expression analysis of ABCA2, CD72, CETN2, KCNG1, NDUFA2, and RPL36AL for various cell types in the brain tissue. No data available about CREBRF in AlzData. [file Image_1.tif]
